# A modular, isolated high-voltage switch for application in ion mobility spectrometry

**DOI:** 10.1016/j.ohx.2024.e00574

**Published:** 2024-08-23

**Authors:** Martin Lippmann, Moritz Hitzemann, Jonas Winkelholz, David Bailey, Stefan Zimmermann

**Affiliations:** Leibniz University Hannover, Institute of Electrical Engineering and Measurement Technology, Department of Sensors and Measurement Technology, Appelstr. 9A, 30167 Hannover, Germany

**Keywords:** Ion mobility spectrometry, High-voltage switch, Ion gating, Isolated gate driver

## Abstract

Ion mobility spectrometry is an emerging technology in trace gas analysis that has moved from typical safety and security applications to many other fields ranging from environmental and food quality monitoring to medicine and life sciences. Nevertheless, further dissemination, including the development of new instruments and the expansion into new fields of application requires the availability of the fundamental components of ion mobility spectrometers. For example, the electronics is essential for the analytical performance, but is only provided by specialized manufacturers due to specific requirements. In this paper, we present a modular, isolated high-voltage switch that can be operated at an isolated potential. The modular design enables tailoring its configuration to the required application. Each module can switch a voltage of up to 3 kV, and can be operated with an offset voltage of up to 7 kV. The switch has rise and fall times of less than 25 ns, making it suitable for a wide range of applications, e.g., in ion mobility spectrometry. Finally, the presented modular, isolated high-voltage switch was used in a push–pull configuration to generate the injection pulse of the ion gate. The new modular, isolated high-voltage switch shows similar performance compared to a commercially available high-voltage switch.

## Graphical abstract

**Figure:**
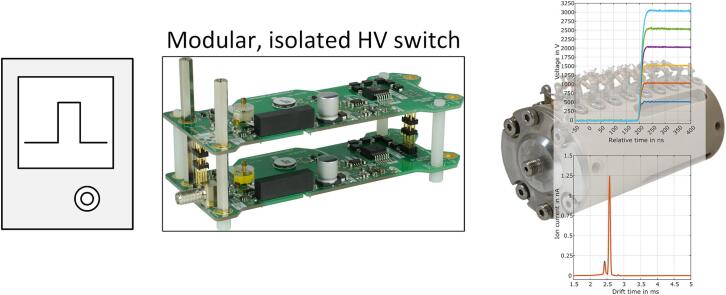

Specifications table

**Table:** 

Hardware name	Modular, isolated high-voltage switch
Subject area	• Chemistry and biochemistry • Biological sciences (e.g., microbiology and biochemistry) • Environmental, planetary, and agricultural sciences • General
Hardware type	• Field measurements and sensors • Electrical engineering and computer science
Closest commercial analog	Custom High-Voltage Switches from companies like “Behlke Power Electronics GmbH”
Open source license	CC BY 4.0
Cost of hardware	Approx. 100 €
Source file repository	https://doi.org/10.17632/gtnv5dwjk5.1

## 1 Hardware in context

Ion mobility spectrometers (IMS) have been used since the 1950s to detect gases at trace levels. Initially, IMS were mainly used in safety and security applications, e.g., for the detection of highly toxic compounds, explosives and drugs of abuse [1], [2], but today, its application spans from environmental and food quality monitoring to medicine and life sciences [1], [2], [3]. The analysis of gaseous samples in drift time IMS is based on the separation of different ion species by their specific ion mobility in an electric field. For this reason, the sample has to be ionized in the first step. The generated ions are transferred into the drift tube, where ions of different species are separated based on their ion mobility. At the end of the drift tube, the ions are detected by a Faraday detector, generating a current, which is then converted into a proportional voltage by a transimpedance amplifier. Recording the ion current over time gives the ion mobility spectrum.

The analytical performance of an IMS is mainly described by its detection limit and resolving power [4]. The resolving power is obtained from the drift time of a specific ion species divided by the full width at half maximum (FWHM) of the corresponding peak in the spectrum. Thus, it represents the separation power. The detection limit, however, represents the concentration at which the ion current related to a certain compound is three times the standard deviation of the noise of the zero signal and thus defines the minimum detectable concentration.

The influence of the mechanical design and operating parameters on the analytical performance of IMS has been extensively described in the literature [4], [5], [6], [7], [8], [9], [10], [11], [12], [13], [14]. Furthermore, several easy-to-manufacture, low-cost designs have been published [15], [16], [17], [18]. Those designs mainly consist of printed circuit boards (PCBs) and differ in using flexible or rigid versions of PCBs. However, the electronics required to operate such IMS is expensive when using commercial devices, which limits the broader application of IMS. For instance, fast switching of high voltages on an isolated electric potential is a major challenge that can only be addressed by few manufacturers, e.g., “Behlke Power Electronics GmbH”. In addition to the mechanical design, the electronics significantly affect the analytical performance of IMS, thinking of the bandwidth and noise of the amplifier or operating the ion gate in a defined temporal sequence.

The electric field used to separate the ions typically results in a drift voltage across the entire drift tube in the kilovolt range and the drift voltage directly influences the resolving power of IMS [19]. Given that the detector and amplifier of an IMS are typically ground referenced, the voltages of the ion gate must be operated at high drift voltage potential. Depending on the type of ion gate and the design of the ionization region, voltages in the range of a few volts up to several hundred volts must be rapidly switched. Typically, the pulse width applied to the ion gate ranges from 5 µs to 350 µs [11]. For example, using a drift tube IMS with a so-called field-switching ion gate, the temporal width of the ion packet is mainly defined by the injection voltage used to transfer the ion packet into the drift tube [7]. Consequently, the injection voltage has an impact on the resolving power. Achieving ultra-high resolving power using a field-switching ion gate requires injection voltages up to the low kilovolt range [20]. As a result, the high-voltage switch required for such injection voltages needs an insulation strength in the kilovolt range to isolate the used drift voltage. At the same time, the high-voltage switch must be capable of switching voltages up to the kilovolt range. These requirements imply that the supply voltage of the driver of the high-voltage switch and the switching signal must be isolated. As mentioned before, the commercial market for switches that meet such requirements is limited to a few specialized manufacturers, thus representing a significant cost factor in IMS. For this reason, we present a low-cost and modular alternative of a high-voltage switch, which is capable of switching several kilovolts and operating at high potential of up to 10 kV above reference potential.

## 2 Hardware description

The modular, isolated high-voltage (HV) switch presented in this work is based on a silicon carbide field-effect transistor (SiC-MOSFET) capable of switching up to 3 kV with rise and fall times of under 25 ns. The SiC-MOSFET is driven by a non-insulated gate driver, which is powered by an isolated power supply and controlled via optical data transmission. The isolated power supply of the gate driver is realized by an inductive power transmission utilizing QI coils, which also provide the power required for switching the SiC-MOSFET [21], [22], [23], [24], [25], [26]. Moreover, the inputs and outputs, as well as the creepage distances of the PCBs, are designed in a way that it becomes possible to stack several modules of the HV switch for switching up to ±9 kV and isolating 10 kV. Additionally, a push–pull stage configuration can be constructed by implementing an inversion of the control signal for switching between two defined potentials.

Therefore, this modular, isolated HV switch is suitable for the following ion mobility spectrometry applications:

• Switching the injection voltage of a field-switching ion gate operated at a potential of ±10 kV above reference potential.
• Fast polarity switching of the drift voltage (up to 9 kV) for an ultra-fast polarity switching IMS, as introduced by Hitzemann et al. [27].
• Switching the drift field in a moving-field IMS introduced by Bohnhorst et al. [28].
• Switching the dispersion voltage of a FAT-IMS introduced by Bohnhorst et al. [29].

In addition, the switch can be used in other applications that need to rapidly switch high-voltages:

• Switching between two potentials up to ±9 kV
• Switching of voltages isolated at a high electrical potential of ±10 kV above reference potential
• Switching loads at high-voltage of up to ±9 kV

Possible other applications include driving electrostatic electrodes, extraction grids, and deflection plates of ion guides, piezos or other capacitive loads. In these cases, a push–pull configuration of two modular, isolated HV switches is recommended. Switching inductive loads using the modular, isolated HV switch is not possible without additional protective measures. Furthermore, the modular, isolated switch is not suitable for high-power applications as the MOSFET is just rated up to 4 A and moreover, the design does not provide sufficient cooling of the MOSFET for these applications.

A further increase in creepage distances on the PCBs and the change of the switching signal's optical path may allow even higher insulation strengths, insulation resistance and higher voltages to be switched.

## 3 Design files summary

All schematics, production files and CAD files, summarized in Table 1 below, can be downloaded from the MendeleyData repository (https://doi.org/10.17632/gtnv5dwjk5.1).

**Table 1:** Design files.

**Design file name**	**File type**	**Open source license**	**Location of the file**
IMS_Stackable_HV_Switch.PrjPcb	Schematics	CC BY 4.0	MendeleyData
IMS_Stackable_HV_Switch.PrjPCBStructure	Schematics	CC BY 4.0	MendeleyData
IMS_Stackable_HV_Switch.Annotation	Schematics	CC BY 4.0	MendeleyData
TopSheet.SchDoc	Schematics	CC BY 4.0	MendeleyData
LowsideConnector.SchDoc	Schematics	CC BY 4.0	MendeleyData
Lowside_RX.SchDoc	Schematics	CC BY 4.0	MendeleyData
IsoPower.SchDoc	Schematics	CC BY 4.0	MendeleyData
IsoPower_FlyBuck.SchDoc	Schematics	CC BY 4.0	MendeleyData
HV_Connection.SchDoc	Schematics	CC BY 4.0	MendeleyData
Highside_RX.SchDoc	Schematics	CC BY 4.0	MendeleyData
GateSegment.SchDoc	Schematics	CC BY 4.0	MendeleyData
HV_Switch_Schematics.pdf	Schematics	CC BY 4.0	MendeleyData
HV_Switch_3D.pdf	3D PCB View	CC BY 4.0	MendeleyData
TopSheet.apr	Gerber Files	CC BY 4.0	MendeleyData
TopSheet.EXTREP	Gerber Files	CC BY 4.0	MendeleyData
TopSheet.GBL	Gerber Files	CC BY 4.0	MendeleyData
TopSheet.GBO	Gerber Files	CC BY 4.0	MendeleyData
TopSheet.GBP	Gerber Files	CC BY 4.0	MendeleyData
TopSheet.GBS	Gerber Files	CC BY 4.0	MendeleyData
TopSheet.GM1	Gerber Files	CC BY 4.0	MendeleyData
TopSheet.GTL	Gerber Files	CC BY 4.0	MendeleyData
TopSheet.GTO	Gerber Files	CC BY 4.0	MendeleyData
TopSheet.GTP	Gerber Files	CC BY 4.0	MendeleyData
TopSheet.GTS	Gerber Files	CC BY 4.0	MendeleyData
TopSheet.REP	Gerber Files	CC BY 4.0	MendeleyData
TopSheet-macro.APR_LIB	Gerber Files	CC BY 4.0	MendeleyData
TopSheet.DRR	NC Drill Files	CC BY 4.0	MendeleyData
TopSheet.LDP	NC Drill Files	CC BY 4.0	MendeleyData
TopSheet-NonPlated.txt	NC Drill Files	CC BY 4.0	MendeleyData
TopSheet-Plated.txt	NC Drill Files	CC BY 4.0	MendeleyData
Assembly_Enclosure.iam	CAD Files	CC BY 4.0	MendeleyData
Enclosure_body.ipt	CAD Files	CC BY 4.0	MendeleyData
Enclosure_cover.ipt	CAD Files	CC BY 4.0	MendeleyData
HV-Switch.ipt	CAD Files	CC BY 4.0	MendeleyData
Enclosure_cover.stl	CAD Files	CC BY 4.0	MendeleyData
Enclosure_body.stl	CAD Files	CC BY 4.0	MendeleyData

## 4 Bill of materials summary

The individual cost for one single modular, isolated HV switch is approximately 100 €. Given that a substantial portion of the costs is attributed to the PCB, the expense diminishes significantly with the production of multiple modular, isolated HV switches, leading to a notable reduction in the costs per switch as the costs per board decrease. For instance, fabricating six modular, isolated HV switches reduces the cost per modular, isolated HV switch below 90 €. Constructing a push–pull configuration serving as a high-voltage switch for voltages of up to 3 kV entails total cost of approximately 225 €. The additional costs, which exceed a mere doubling, arise primarily from including connectors between the two PCBs. A comprehensive breakdown of the materials is provided in Table 2.

**Table 2:** Bill of materials used for this hardware.

**Designator**	**Component**	**Number**	**Cost per unit-currency**	**Total cost-currency**	**Source of materials**
Stackable HV switch unit PCB	Printed Circuit Board	1	14,80 €	14,80 €	https://www.multi-circuit-boards.eu
C1, C3, C5, C9, C10, C17, C22, C28	Capacitor SMD 0805 0.1uF 50 V X7R	8	0,15 €	1,18 €	https://de.farnell.com/tdk/cga4j3x7t2e104k125aa/kondensator-0-1uf-0805-250v-10/dp/2211001
C2, C4, C6, C7, C11, C15, C18, C24, C25	Capacitor SMD 0805 1uF 50 V X7R	9	0,18 €	1,65 €	https://de.farnell.com/kemet/c0805c105k5ractu/kondensator-1uf-50v-x7r-0805/dp/2118131?Ntt=2118131
C8	Capacitor SMD 0805 56pF 50 V NP0	1	0,07 €	0,07 €	https://de.farnell.com/yageo/ac0805jrnpo9bn560/kondensator-56pf-50v-mlcc-0805/dp/4166211
C12, C21, C26, C27, C33	Capacitor SMD 0805 10uF 35 V X5R	5	0,21 €	1,07 €	https://de.farnell.com/murata/grm21br6ya106ke43l/kondensator-10-f-35v-10-x5r-0805/dp/2611943
C13	Capacitor SMD 0805 220pF 50 V NP0	1	0,11 €	0,11 €	https://de.farnell.com/murata/grm21a5c2e221jw01d/kondensator-220pf-250v-mlcc-0805/dp/3340793
C14, C20	Capacitor SMD 0805 10nF 50 V NP0	2	0,12 €	0,24 €	https://de.farnell.com/murata/grm2195c1h103ja01d/kondensator-0-01-f-50v-5-c0g-np0/dp/8820074
C16	Capacitor SMD 0805 1.2nF 50 V NP0	1	0,05 €	0,05 €	https://de.farnell.com/murata/grm2165c2a122ja01d/kondensator-1200pf-100v-mlcc-0805/dp/3581389
C19	SMD Panasonic Electrolyt-Capacitor 68uF 50 V	1	2,13 €	2,13 €	https://www.mouser.de/ProductDetail/Panasonic/50SVPF68M?qs=OE1iw1LrrPHIF78tf5kBjw%3D%3D
C31	Trimmer BFC2808 vertical series 5.5 pF – 65 pF	1	5,74 €	5,74 €	https://www.mouser.de/ProductDetail/Vishay-BC-Components/BFC280831659?qs=rCu%252BC%252BJkvKa%252Bs3bgN0PQyg%3D%3D
D1, D2, D3, D4, D6, D7	Diode SMD P6SMB550A	6	0,45 €	2,68 €	https://www.mouser.de/ProductDetail/Bourns/P6SMB550A?qs=QB0ovJDgFgu%252BWplPxxsFbg%3D%3D
D8, D9, D10, D11, D13, D14	Diode SMD 1N4148W	6	0,09 €	0,56 €	https://www.mouser.de/ProductDetail/Diotec-Semiconductor/1N4148W?qs=OlC7AqGiEDmsbkbAeXQtXg%3D%3D
D5	LED SMD 0805 yellow	1	0,12 €	0,12 €	https://www.reichelt.de/led-smd-2012-0805-gelb-8-mcd-120--smd-led-0805-ge-p31437.html?GROUPID=3035&&r=1
D12	Diode SMD P6SMB30A	1	0,38 €	0,38 €	https://de.farnell.com/littelfuse/p6smb30a/tvs-diode-600w-25-6v-unidir-do/dp/2679614?st=p6smb30a
D15, D16	LED SMD 0805 green	2	0,12 €	0,24 €	https://www.reichelt.de/smd-led-0805-2012-super-gruen-15-mcd-120--smd-led-0805-gn-p31436.html?GROUPID=3035&&r=1
IC1	Non-inverting FET driver IXDN609SI	1	2,79 €	2,79 €	https://de.farnell.com/ixys-semiconductor/ixdn609si/mosfet-treiber-40-bis-125-c/dp/3580495?st=ixdn609si
IC2	Dual Schmitt trigger SN74LVC2G14	1	0,55 €	0,55 €	https://www.mouser.de/ProductDetail/Texas-Instruments/SN74LVC2G14IDCKRQ1?qs=PVVDbbWpW3LjDJNCpwWTow%3D%3D
OPK1	Optocoupler OPI1268	1	7,74 €	7,74 €	https://www.mouser.de/ProductDetail/Optek-TT-Electronics/OPI1268?qs=NVJATC80C4%252BwL4AEB9cmsw%3D%3D
P9, P11	Board-to-Board Connector 3X2	2	1,89 €	3,78 €	https://de.farnell.com/wurth-elektronik/61000621821/steckv-buchsenleiste-6p-2reihen/dp/2827866
P8, P10	Board-to-Board Connector 3X2	2	1,10 €	2,20 €	https://de.farnell.com/wurth-elektronik/61000621121/stiftleiste-2-54mm-smd-6pol/dp/2356218?ost=61000621121
P10	SMA print connector	1	3,95 €	3,95 €	https://de.farnell.com/te-connectivity/5-1814400-2/hf-koax-steckverbinder-abg-sma/dp/3364890
Q1	SiC-MOSFET G2R1000MT33J	1	17,38 €	17,38 €	https://de.farnell.com/genesic-semiconductor/g2r1000mt33j/mosfet-sic-n-kanal-3-3kv-4a-74w/dp/3598638
Q2, Q3	MOSFET N-channel IRLML6346	2	0,34 €	0,67 €	https://www.mouser.de/ProductDetail/Infineon-IR/IRLML6346TRPBF?qs=9%252BKlkBgLFf3TGIqhHXU%2FwA%3D%3D
R1	Resistor SMD 0805 4.7 Ohm	1	0,02 €	0,02 €	https://de.farnell.com/vishay/crcw08054r70fkea/dickschichtwiderstand-4r7-1-0/dp/1653014RL
R2, R17, R19	Resistor SMD 0805 10 K	3	0,03 €	0,08 €	https://de.farnell.com/vishay/rca080510k0fkea/dickschichtwiderstand-10k-1-0805/dp/2616592
R8	Resistor SMD 0805 75 K	1	0,03 €	0,03 €	https://de.farnell.com/vishay/crcw080575k0fkea/dickschichtwiderstand-75k-1-0/dp/1653035
R9, R13	Resistor SMD 0805 910 K	2	0,04 €	0,07 €	https://de.farnell.com/vishay/crcw0805910kfkea/dicksch-widerstand-910k-1-0-125w/dp/2139117
R4	Resistor SMD 0805 100 K	1	0,03 €	0,03 €	https://de.farnell.com/vishay/crcw0805100kfkea/dicksch-widerstand-100k-1-0-125w/dp/1469860
R12	Resistor SMD 0805 510 K	1	0,04 €	0,04 €	https://de.farnell.com/vishay/crcw0805510kfkea/dicksch-widerstand-510k-1-0-125w/dp/2139087
R14	Resistor SMD 0805 1 Meg	1	0,03 €	0,03 €	https://de.farnell.com/vishay/crcw08051m00fkea/dickschichtwiderstand-1m-1-0-125w/dp/1652946
R15	Resistor SMD 0805 4.7 K	1	0,03 €	0,03 €	https://de.farnell.com/vishay/crcw08054k70fkea/dickschichtwiderstand-4k7-1-0/dp/1469923
R11	Resistor SMD 0805 75 R	1	0,03 €	0,03 €	https://de.farnell.com/vishay/crcw080575r0fkea/dickschichtwiderstand-75r-1-0/dp/1469955
R7	Resistor SMD 0805 1 K	1	0,03 €	0,03 €	https://de.farnell.com/vishay/crcw08051k00fkea/dickschichtwiderstand-1k-1-0-125w/dp/1469847
R10	Resistor SMD 0805 51 K	1	0,03 €	0,03 €	https://de.farnell.com/vishay/crcw080551k0fkea/dickschichtwiderstand-51k-1-0/dp/1653018
R16, R18	Resistor SMD 0805 100 Ohm	2	0,03 €	0,05 €	https://de.farnell.com/vishay/crcw0805100rfkeb/widerstand-100r-1-0-125w-0805/dp/4177711
R20	Resistor SMD 0805 220 K	1	0,02 €	0,02 €	https://de.farnell.com/multicomp/mcwf08p2203ftl/dickschichtwiderstand-220k-1-0/dp/2694120
U2	Step-Down Controller LM3150MH	1	3,11 €	3,11 €	https://www.ti.com/product/LM3150#order-quality
U3	Inverting Charge Pump LTC3260	1	9,12 €	9,12 €	https://www.mouser.de/ProductDetail/Analog-Devices/LTC3260EDEPBF?qs=hVkxg5c3xu9aeneAPtdUAA%3D%3D
U1	LDO 3.3 V TPS70933	1	1,21 €	1,21 €	https://www.mouser.de/ProductDetail/Texas-Instruments/TPS70933DBVR?qs=Z9twEOuL%252B%2FJOaGO60BtvLA%3D%3D
QI1, QI2	Qi-Inductor Würth Electronic 18 uH, 0.7 A, 0.5 Ohms	2	7,97 €	15,94 €	https://www.mouser.de/ProductDetail/Wurth-Elektronik/760308101208A?qs=6mGTcvmWjFtRJtuJp2qBjQ%3D%3D
				**99,91 €**	

optional optical connection:					
LWL1	LWL-Receiver AFBR-2624Z	1	22,79 €	22,79 €	https://www.mouser.de/ProductDetail/Broadcom-Avago/AFBR-2624Z?qs=60YbxKv0rtDJIJtl89oIpg%3D%3D

## 5 Build instructions

**Fig. 1:**
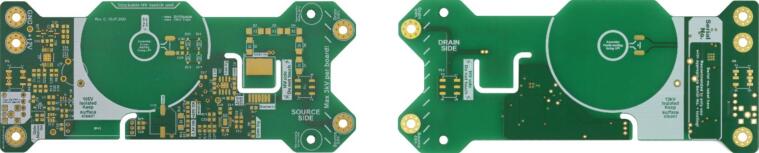
Printed circuit board of the modular, HV switch showing the top side (left) and bottom side (right).

The PCB of the modular, isolated HV switch and the soldering stencil can be ordered from a PCB manufacturer using the Gerber files from MendeleyData. For the ordering process, the PCB board size (140.97 mm × 59.69 mm), the PCB thickness (1.55 mm), and the copper thickness (35 µm) are essential parameters. It is also useful to choose an overlay silk screen for both sides in the ordering process since this reduces the effort of assembly and is also helpful during the functional test. Fig. 1 shows the manufactured PCB, with the top side depicted on the left and the bottom side on the right. When ordering the stencil, it is advisable to request dimensions at least 20 mm wider (160.87 mm × 79.69 mm) to facilitate the assembly process.

**Fig. 2:**
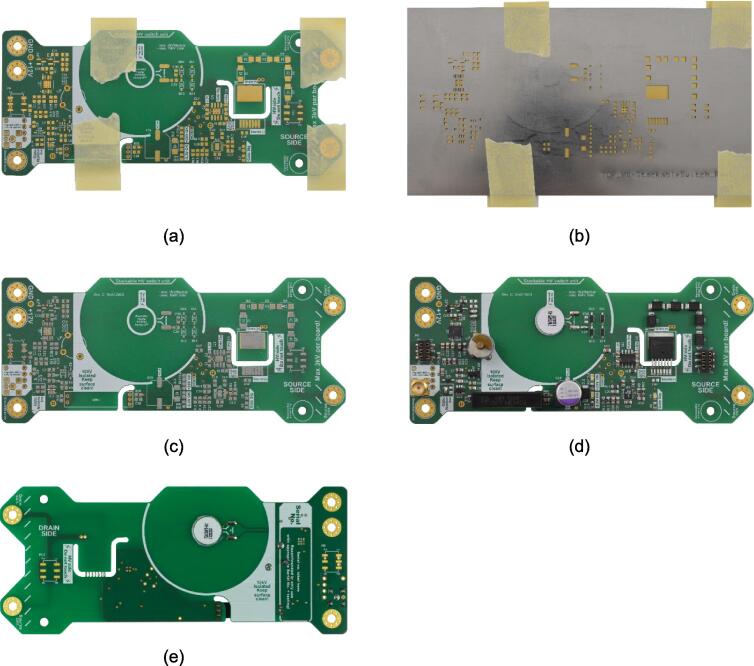
Assembly sequence of the printed circuit board (PCB) of the modular, high-voltage switch. a) PCB top side fixed with masking tape, b) Stencil fixed on the PCB with masking tape, c) top side of the PCB with applied solder paste, d) top side of the PCB ready assembled, e) bottom side of the PCB ready assembled.

Fig. 2 shows the assembly process, outlining the outcome of each step. It is advisable to start by placing the Surface-Mounted Devices (SMD) on the top side of the PCB. The necessary components are described in the bill of material and must be placed on the corresponding pads of the PCB. The individual component position on the PCB is shown by the overlay silk screen printed on the PCB and in the 3D representation uploaded on MendeleyData.

For this phase, soldering paste and either a hot air station or a soldering oven are essential to solder the Integrated Circuits (ICs) featuring a dedicated ground plane under the package (U3.3 LM3150MH Texas Instruments, U3.4 LTC3260 Analog Devices, IC1.1 MCP1406 Microchip Technology). To begin with, the PCB is attached to a smooth surface e.g. cardboard with masking tape, and the top side's soldering stencil is attached and fixed with masking tape, as shown in Fig. 2a and b. Subsequently, the solder paste (e.g. CHIP QUIK SMD4300SNL10) is applied to the pads of the components with a squeegee (an old plastic card e.g. credit card works fine as well) and the stencil can be removed carefully by lifting it evenly to minimize smearing of the solder paste. This process should yield a PCB prepared for placement of the components as shown in Fig. 2c.

When positioning the SMD components, it is recommended to start with the ICs and MOSFETs before progressing to the capacitors, resistors, and diodes. Subsequently, the PCB with the SMD components placed can be soldered in a soldering oven or with a hot air station. Once the SMD components are soldered, the through-hole (THT) components located on the top side can be soldered using a standard soldering iron and lead-free solder. The finalized PCB is shown in Fig. 2d and e.

**Fig. 3:**
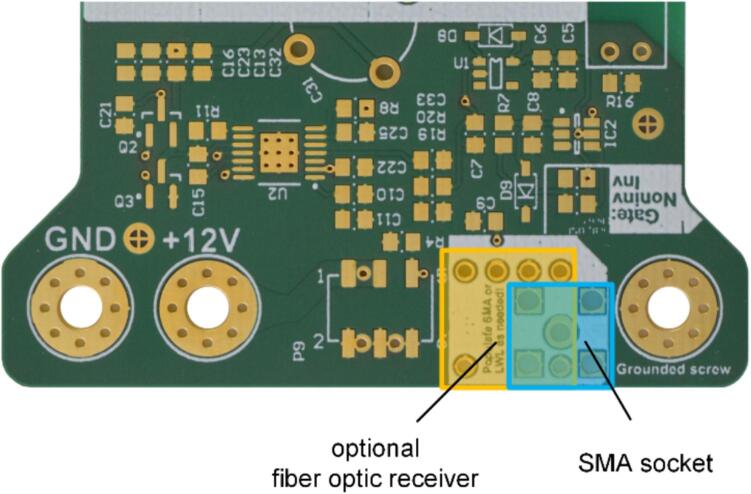
Section of the board with combined footprint for using an SMA socket (5–1814400-2 TE Connectivity) (blue) as signal input or optional use of a fiber optic receiver (AFBR-2624Z Broadcom) (orange). (For interpretation of the references to colour in this figure legend, the reader is referred to the web version of this article.)

To configure multiple modular, isolated HV switches in a push–pull setup or to operate the switches with higher switching voltages, it is crucial to solder the SMA print socket (P4.12) onto only one of the PCBs. Conversely, if it is intend to control the boards with a fiber optic cable, an optional fiber optic receiver (LWL4.1) needs to be soldered instead of the SMA print socket, as depicted in Fig. 3.

**Fig. 4:**
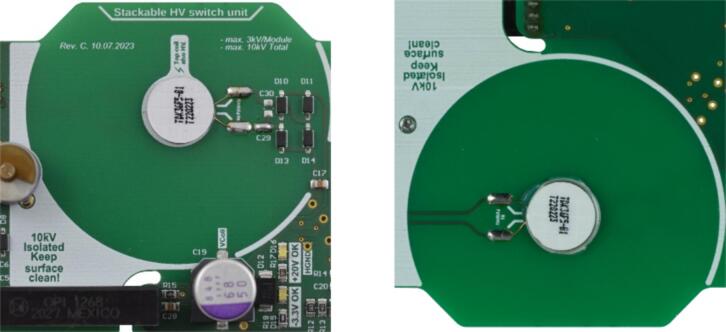
QI Coils assembled on the printed circuit board (PCB), showing the top side (left) and the bottom side (right). The ferrite on the coils faces away from the PCB, and the wires are soldered to the respective pads.

To complete the assembly, the QI coils (QI1, QI2) need to be installed. Therefore, apply 2 K-epoxy glue (such as UHU Endfest 300) at the center of the inner circle on both the top and bottom sides of the board. Ensure that the ferrite foil of the coil faces away from the PCB during installation. Subsequently, solder the supply cables of the QI coils to the respective pads using lead-free solder. The correct positioning of the coils is shown in Fig. 4 for both the top side (left) and the bottom side (right).

Finally, depending on operating mode of the modular, isolated HV switch, the inversion of the gate signal has to be defined. Table 3 shows the application scenarios and which inversion has to be selected. For example, if the modular, isolated HV switch is used as a low-side switch, active-high, the solder bridge must be set at ‘Noninv’. A push–pull configuration needs at least one low-side and one high-side switch.

**Table 3:** Gate signal inversion mode depending on the use-case of the modular, high-voltage switch.

**Configuration**	**Gate signal inversion**
Single switch – active high	‘Noninv’
Single switch – active low	‘Inv’
Push-pull	Upper switch: ‘Noninv’; Lower switch: ‘Inv’
Push-pull – inverted	Upper switch: ‘Inv’; Lower switch: ‘Noninv’

Prior to testing the modular, isolated HV switch, it is of utmost importance to ensure that the PCBs are free of solder and flux residues. In particular, the area around the QI coils must be clean to prevent voltage flashovers due to increased conductivity caused by residues. The cleaning process can be accomplished using isopropyl alcohol and deionized water.

Before initial operation, the PCB must be visually checked for possible soldering faults and misplaced or missing components. If no solder bridges between pins or pads of the ICs or other components are evident, the resistance between +12 V supply voltage and GND should be measured with a multimeter. A resistance of approximately 290 kΩ (measured with a Keysight 34465A) indicates proper functionality. When connecting a 12 V supply voltage, the current consumption should be about 110 mA to 170 mA and the green LED (D11) should light up. This indicates correct operation of the IC controlling the QI coil and the presence of the voltage on the isolated side. Next, measure the supply voltage on the isolated side between the marked HGND and +20 V and −3.3 V. Slight adjustment of the tunable capacitor C31 using a plastic screwdriver may be necessary to optimize the voltage, aiming for a range between 18 V to 22 V with an optimal voltage around 20 V. Additionally, check the −3.3 V voltage to ensure proper operation.

Subsequently, apply a static input signal of either ‘0 V’ or ‘3.3 V’ to the SMA print socket to test the full functionality of the modular, isolated HV switch. Depending on the inversion of the gate, the SiC-MOSFET will either conduct or block with a logic ‘0′ or ‘1′ input, respectively. Verify the SiC-MOSFET's state with a multimeter connected between the drain and source terminals, ensuring that the resistance in the conducting state is around 1.3 Ω and in the blocking state exceeds 1.1 GΩ (measured with a Keysight 34465A). Upon successful completion of all tests, the board is ready for operation.

**Fig. 5:**
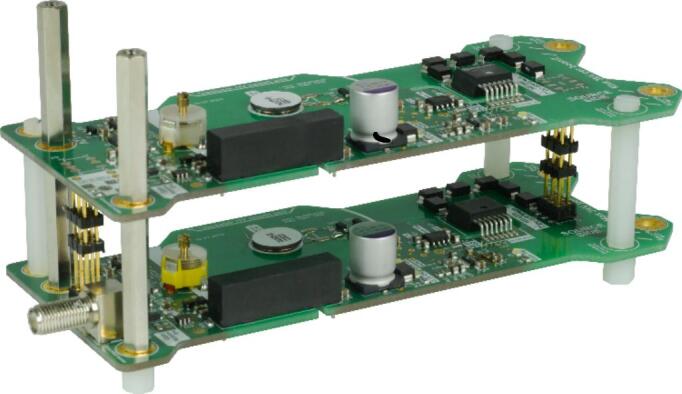
Push-pull configuration of two modular, high-voltage switches. The supply voltage, the control signal, the drain contact of the upper side, and the source contact of the lower side are connected via the headers between the printed circuit boards. The output signal can be taken either at the drain contact of the upper side or at the source contact of the lower side.

Depending on the desired switching voltage, multiple modular high-side HV switches may be stacked on top of each other to achieve the necessary dielectric strength. Each individual module has the capability to switch up to 3 kV. For instance, utilizing six modular, isolated HV switches stacked in a push–pull configuration enables switching of a maximum voltage of ±9 kV. An exemplary push–pull configuration containing one high-side switch and one low-side switch is shown in Fig. 5.

**Fig. 6:**
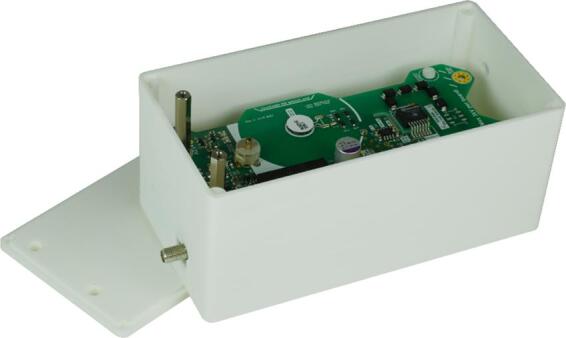
Push-pull stage of the HV switch in 3D-printed housing.

Finally, Fig. 6 displays the push–pull stage of the HV switch enclosed within the 3D-printed housing built from ABS material using a MakerBot METHOD X 3D printer. This housing incorporates specific cutouts tailored for high-voltage connections, switching signals, and power supply inputs of the modular, isolated HV switch. Notably, it serves to safeguard against inadvertent contact with high-voltage components.

*Safety instructions:*

*Soldering pastes and solder are potentially hazardous to health and environment and soldering fumes are toxic, so the PCB should only be assembled using appropriate personal protective equipment and measures. Care should also be taken with the waste since the solder paste and other electrical components can contain substances that are harmful to the environment. When using PCB cleaning agents, suitable personal protective equipment and measures should be used, as the substances used for this purpose may also be hazardous to health.*

*The functional tests have to be performed by trained staff and without high voltage until proper function is ensured.*

## 6 Operation instructions

**Fig. 7:**
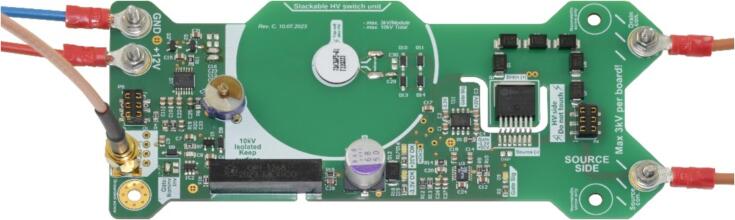
Exemplary connected high-voltage switch, with the 12 V power supply on the left side red (+) and blue (GND) cable. The control signal is connected via the SMA cable (left bottom side) and connected to a function generator. The high-voltage is connected via the two brown cables (right side). (For interpretation of the references to colour in this figure legend, the reader is referred to the web version of this article.)

For operation, a 12 V supply voltage must be connected to the two mounting holes on the left side, as depicted in Fig. 7. Subsequently, the source and drain can be connected with suitable cables, rated for the voltage used, to the mounting holes on the high-voltage side. Ensuring that the high-voltage source is turned off and de-energized before working on these cables and terminals is imperative.

**Fig. 8:**
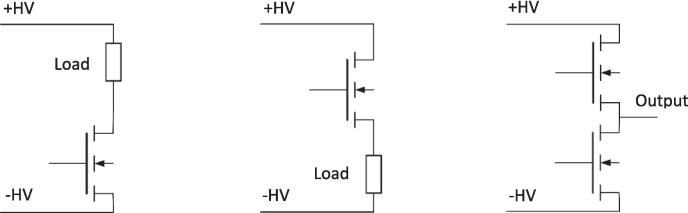
Modular, isolated high-voltage switch used as a low-side switch (left), a high-side switch (center) or in push–pull configuration (right).

Fig. 8 shows the wiring of the modular, isolated HV switches for three different application scenarios. From the left to right, the wiring represents a low-side switch, a high-side switch, and a push–pull stage. If the modular high-voltage switch shall be used as a low-side switch, the source connector has to be connected to the lower electric potential and the drain connector to the low side of the load. If used as a high-side switch, the high electric potential is connected to the drain connector, while the source connector is connected to the high side of the load. In push–pull configuration, the drain side of the high-side switch is connected to the higher electrical potential + HV, while the lower electric potential −HV is connected to the source side of the low-side switch. The source connector of the high-side switch is connected to the drain connector of the low-side switch so that the potential can be switched between the two voltages +HV and −HV. Each modular, isolated HV switch contains a dead time circuit, which ensures fast turn-off times and slower turn-on times of the MOSFETs and prevents a short circuit from +HV to −HV in a push–pull configuration. The dead time can be varied by changing the resistor R7 and the capacitor C8. For example, increasing this resistor and capacitor further increases the dead time by delaying the MOSFETs' turn-on.

*Safety considerations: Extreme attention is required when operating the modular, isolated high-voltage switch, as the electric potentials can be lethal. Therefore, the electronics presented here must only be used under the mandatory protective measures for electronics, in particular for high-voltage electronics, and should only be operated by appropriately trained personnel. If working on the hardware, the high-voltage plugs must be disconnected, and the power supply must be turned off and de-energized! The authors accept no liability for any work related to the modular, isolated high-voltage switch.*

## 7 Validation and characterization

To validate and characterize the modular, isolated HV switch, we investigated the rising and falling edges of a push–pull stage utilizing two modular, isolated HV switches. The modular, isolated HV switches were powered by a laboratory power supply (erfi Dreifach Netzgerät H26.692), and a 10 kHz TTL signal with a 50 % duty cycle generated by a function generator (Rigol DG4062) was applied to the switching input via an SMA cable. The high-voltage was generated by a high-voltage power supply (FuG HCE 350-3500) and buffered by three 0.047 µF high-voltage capacitors (WIMA FKP1) and one 0.1 µF high-voltage capacitor (WIMA FKP1) in parallel. The output was directly connected to a high-voltage probe (Keysight 10076C) with an input resistance of 66.7 MΩ and an input capacitance of 3 pF. The voltage was measured using an oscilloscope (Keysight Infinii Vision DSOX4104A). A high-voltage current measurement was performed with a current probe (Agilent 1147B).

**Fig. 9:**
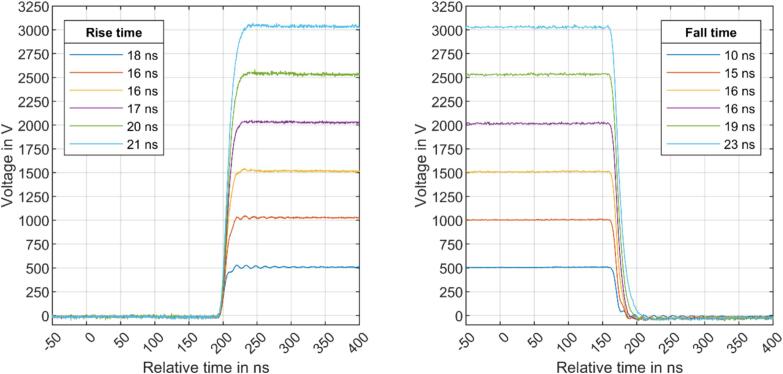
Rising (left) and falling (right) edges using different drain-source voltages.

The rising edges and falling edges for six different voltages are shown in Fig. 9. All voltage curves are plotted against the time scale of the trigger signal. Notably, both the rising edges and the falling edges exhibit a delay of approximately 200 ns, primarily attributed to the propagation delay of the optocoupler used for the switching signal. The maximum rated propagation delay of this optocoupler is 200 ns, while the propagation delays of additional components such as the gate driver or the MOSFET itself are negligible. From the voltage curves, the rise time (10 %–90 %) and the fall time (90 %–10 %) for each of the six different switching voltages were measured. Remarkably, the determined times are well below 25 ns for all voltages without any significant overshoot or undershoot.

Additionally, measuring the insulation resistance with the Fluke 1555 at the maximum output voltage of 10.5 kV resulted in an insulation resistance exceeding 2 TΩ.

**Fig. 10:**
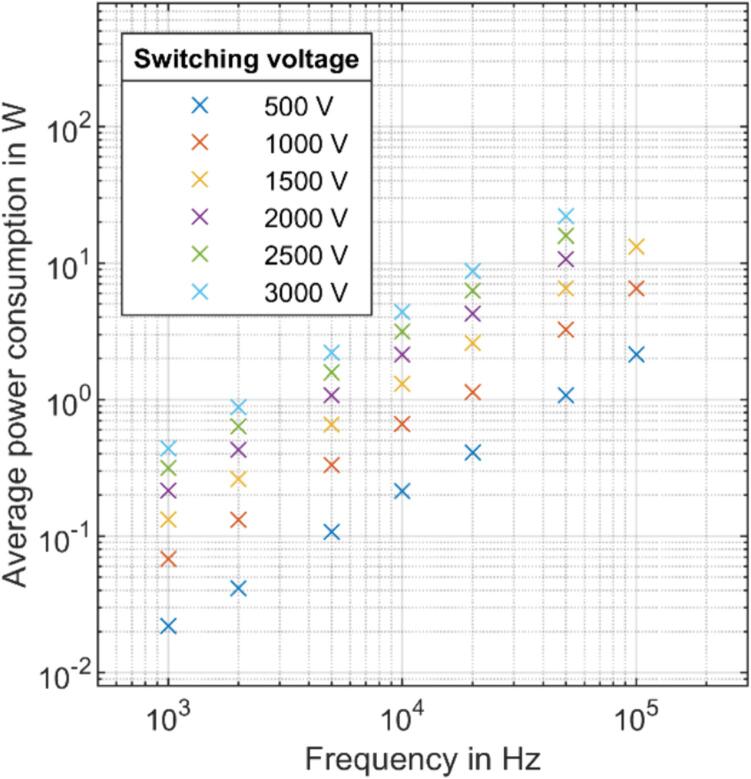
Average power consumption of the push–pull stage from the high-voltage power supply without additional external load at the output except for the directly connected high-voltage probe.

The average power consumption of the push–pull stage from the high-voltage power supply with just the high-voltage probe at the output measured at a switching frequency of 10 kHz and a switching voltage of 500 V is around 20 mW and varies with frequency and switching voltage, as shown in Fig. 10. As expected, the average power consumption of the push–pull stage from the high-voltage power supply is nearly linearly dependent on the switching frequency and the switching voltage. For switching voltages below 1000 V, continuous operation is possible up to frequencies of 100 kHz. For voltages above 1000 V, a continuous operation is only possible up to 20 kHz as the SiC-MOSFETs would otherwise overheat due to the power dissipated during switching. However, higher switching frequencies can be used in a burst mode or by adding a cooler. A rough estimation of the power converted in the high-voltage probe suggests that most of the power consumed from the high-voltage power supply is converted as switching losses in the push–pull stage. It should be noted, however, that the switching losses in the push–pull stage increase further with lower load impedance, and thus, the possible continuous operating point shifts to lower frequencies or voltages for thermal limitations.

**Fig. 11:**
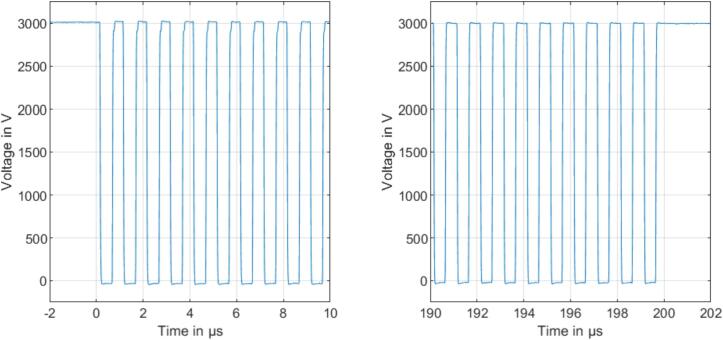
First ten pulses of a burst of 200 pulses with a switching frequency of 1 MHz with 50 % duty cycle (left) and the last ten pulses of the same burst (right).

Fig. 11 shows an excerpt of a burst of 200 pulses at a switching frequency of 1 MHz with a duty cycle of 50 % at a period of 100 ms. The left plot shows the first ten pulses, while the right plot shows the last ten pulses of one exemplary burst. This demonstrates that the push–pull stage consisting of two modular, high-voltage switches, switches the voltage over the entire burst as expected.

The modular, isolated HV switch was then exemplarily used to inject an ion packet into the drift tube of an ion mobility spectrometer. For this experiment, a modified version of our previously reported ultra-high resolution drift tube IMS [11], [20], [30] was used but shortened to a drift length of just 40 mm, as it was available from another project. Its operational parameters are shown in Table 4. It should be noted that this experiment is intended to demonstrate the performance of the modular, isolated HV switch compared to a commercial alternative and the modular, isolated HV switch has not been specially adapted for this application and optimized in size to match with compact IMS setups.

**Table 4:** Operational parameters of the ion mobility spectrometer.

**Parameter**	**Value**
Drift length	40 mm
Drift region diameter	21 mm
Drift field	75 V/mm
Injection field	227 V/mm
Initial ion packet width	20 µs
Repetition rate	40 Hz
Drift gas flow	150 ml/min
Sample gas flow	10 ml/min
Dew point of drift gas and sample gas	−90 °C
Operating pressure	1025 hPa
Operation temperature	25 °C

**Fig. 12:**
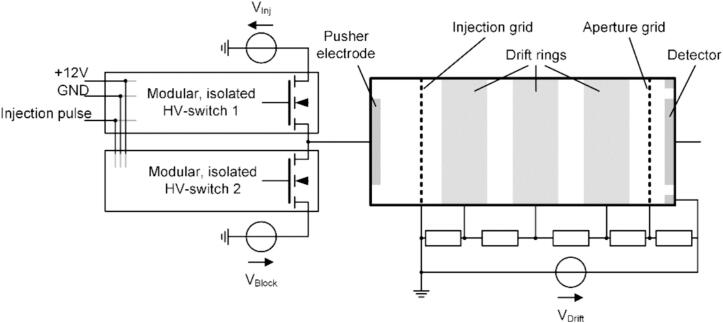
Schematic drawing of the experimental setup using a push–pull configuration of two modular, isolated high-voltage switches to control the ion gating of an ion mobility spectrometer.

A high-voltage power supply (FuG HCP 35-12500) generated the drift voltage. The injection voltage was generated by a high-voltage power supply (FuG HCP 35-1250) while the blocking voltage was supplied by a laboratory power supply unit (Rohde&Schwarz HMP4040). The modular, isolated HV switch was controlled by a self-built data acquisition system [31] and used in a push–pull configuration to switch the output between the injection voltage and the blocking voltage. For comparison, a commercial high-voltage switch (Behlke HTS 91-01-HB-C) was used. For current amplification, a self-built amplifier [32] with a gain of 3.2 GΩ and a bandwidth of 22.6 kHz was used. Purified air with a dew point of −90 °C was used as drift gas and sample gas. Fig. 12 shows a schematic drawing of the experimental setup used for controlling the ion gate of an ion mobility spectrometer.

**Fig. 13:**
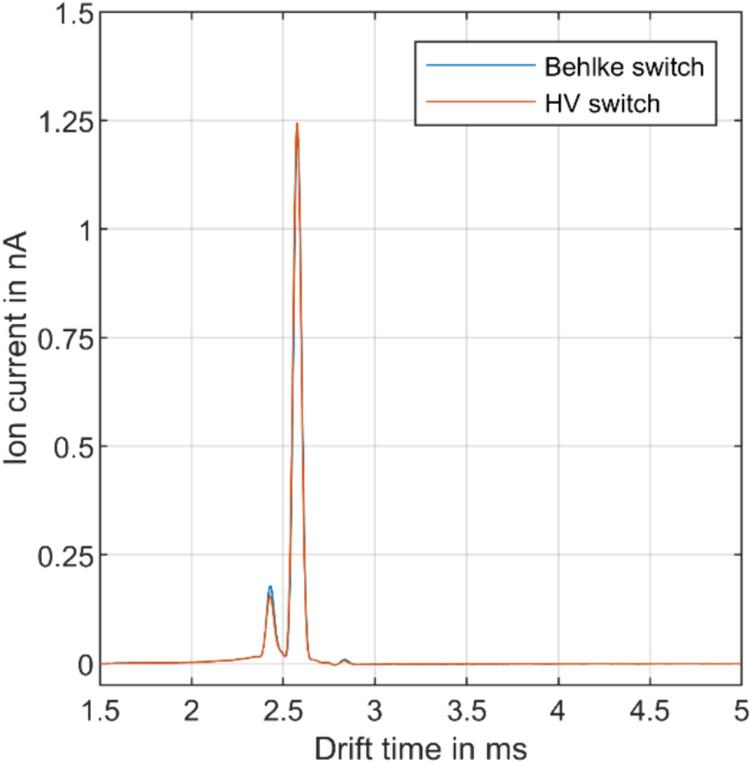
IMS spectra comparing a Behlke switch and the modular, isolated HV switch.

Fig. 13 displays the ion current measured at the detector of the ion mobility spectrometer plotted against the drift time. The measurements for both switches are identical, and the resolving power for the reactant ion peak is *R_P_=50* in both spectra, which is consistent with theoretical predictions for this operating point. The amount of charge measured at the detector is 80 fC for both measurements. As shown in Fig. 13, in the relevant section of the IMS spectrum from 1.5 ms, both switches do not induce any interfering signals, which could disturb the measurement, and no additional shielding measures are required. This demonstrates that the HV switch is a viable replacement for commercially available high-voltage switches in this application.

Overall, the modular, isolated HV switch presented in this work is a cost-effective way of pulsing high-voltages at high potential without relying on expensive special electronics from specialized manufacturers.

## CRediT authorship contribution statement

**Martin Lippmann:** Writing – original draft, Visualization, Validation, Methodology, Investigation, Formal analysis, Data curation, Conceptualization. **Moritz Hitzemann:** Writing – review & editing, Visualization, Validation, Methodology, Investigation, Conceptualization. **Jonas Winkelholz:** Writing – review & editing, Methodology, Investigation. **David Bailey:** Investigation, Conceptualization. **Stefan Zimmermann:** Writing – review & editing, Supervision, Resources, Project administration, Funding acquisition, Conceptualization.

## Declaration of competing interest

The authors declare that they have no known competing financial interests or personal relationships that could have appeared to influence the work reported in this paper.
