# Supplementary figures and images for: A modular, isolated high-voltage switch for application in ion mobility spectrometry
Source: HardwareX. 2024 Aug 23;19:e00574. doi: 10.1016/j.ohx.2024.e00574 (PMC11405904; doi:10.1016/j.ohx.2024.e00574)

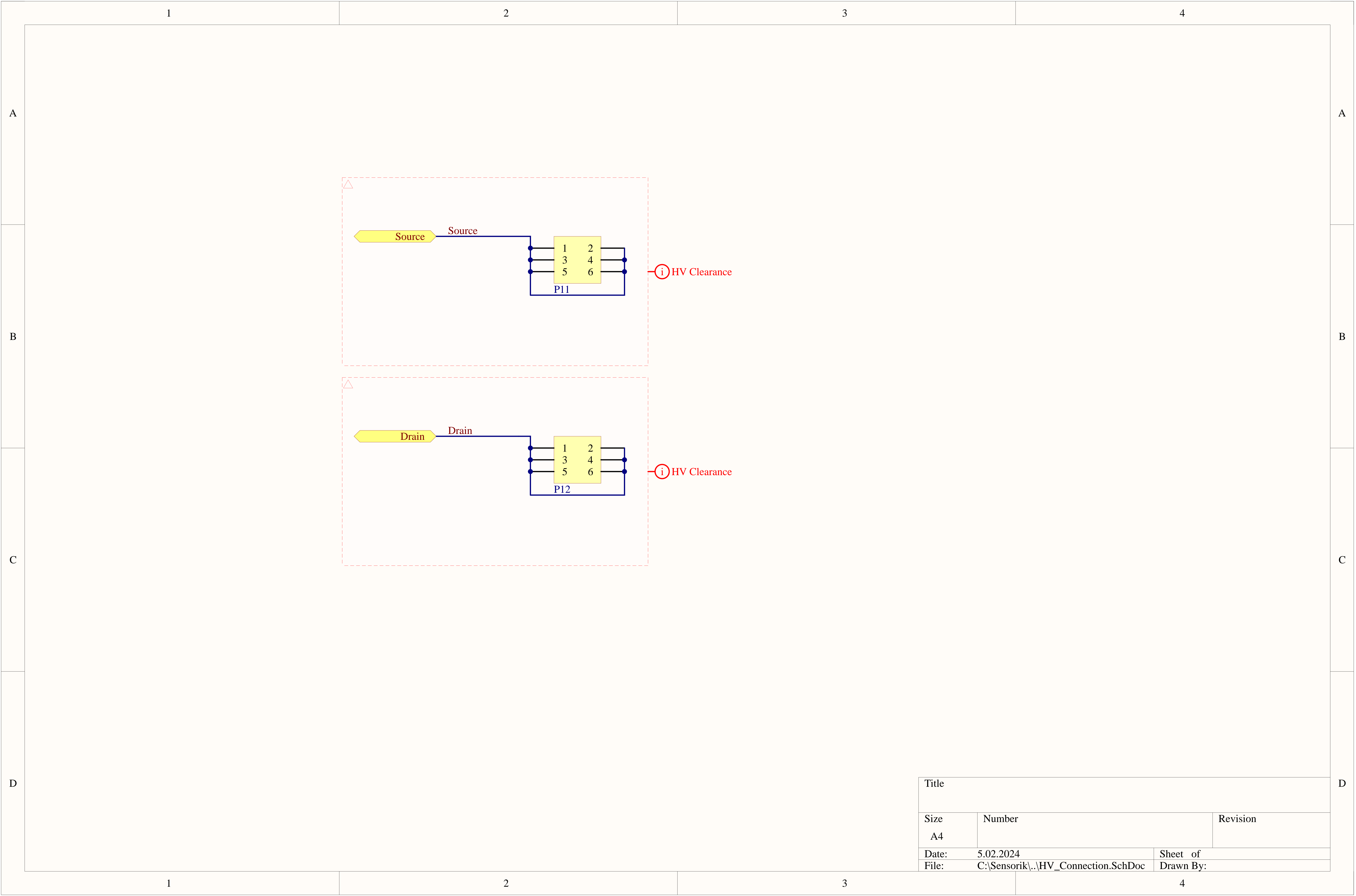





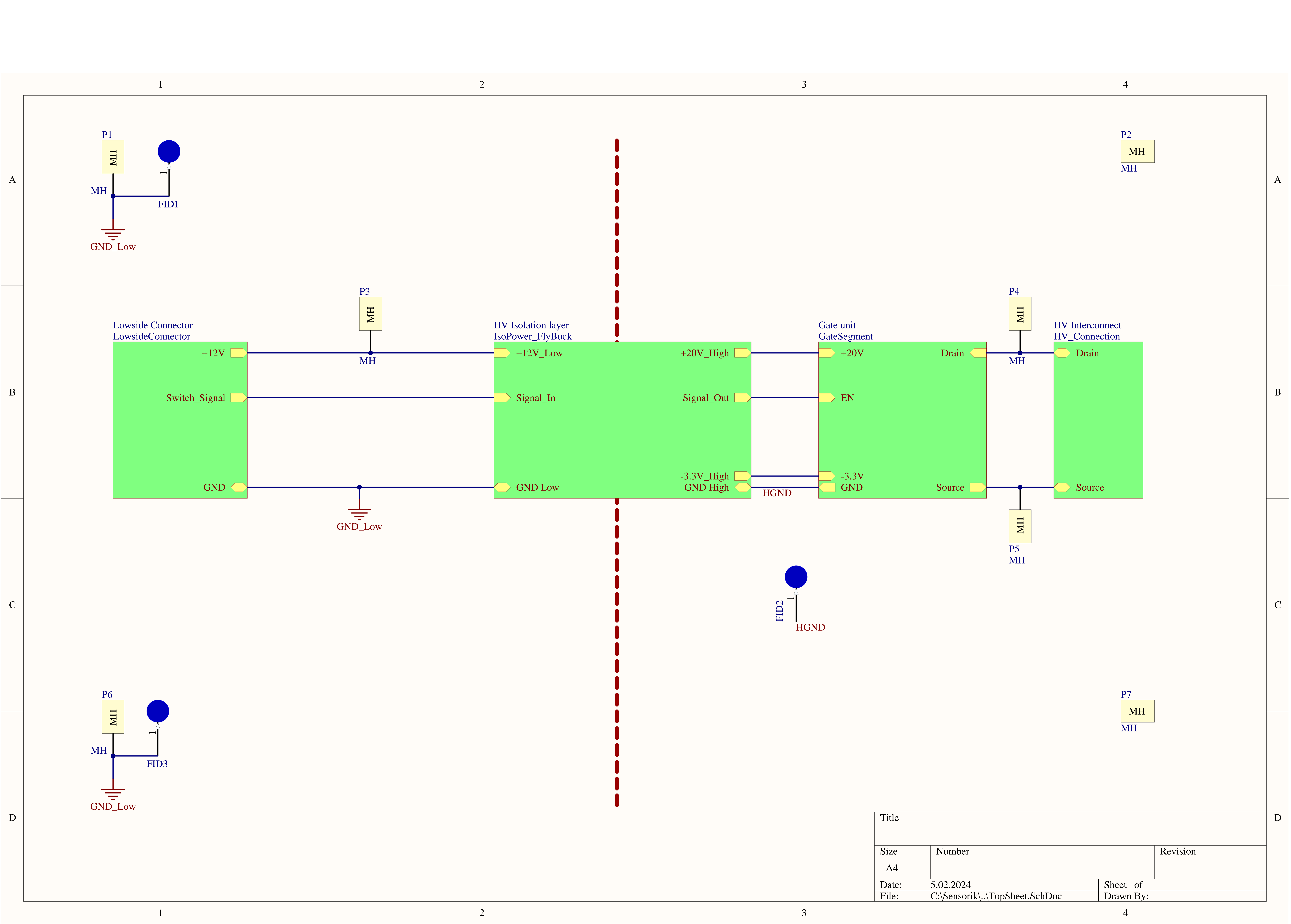

| Title |                                 |           |
|-------|---------------------------------|-----------|
| Size  | Number                          |           |
| A4    | Revision                        |           |
| Date: | 5.02.2024                       | Sheet of  |
| File: | C:\Sensorik\...\TopSheet.SchDoc | Drawn By: |

Supplement: Supplementary Data 3 [file mmc3.pdf]
